# A High Daptomycin Dose Is Associated with Better Bacterial Clearance in Infections Caused by Vancomycin-Resistant Enterococcus faecium Regardless of Daptomycin Minimum Inhibitory Concentration in a Rat Infective Endocarditis Model

**DOI:** 10.1128/spectrum.02551-22

**Published:** 2022-10-03

**Authors:** Jann-Tay Wang, Chia-Jui Yang, Jia-Ling Yang, Shu-Wen Lin, Yu-Chung Chuang, Wang-Huei Sheng, Yee-Chun Chen, Shan-Chwen Chang

**Affiliations:** a Department of Internal Medicine, National Taiwan University Hospital, Taipei, Taiwan; b Department of Internal Medicine, Far Eastern Memorial Hospital, New Taipei City, Taiwan; c School of Medicine, National Yang Ming Chiao Tung University, Taipei, Taiwan; d School of Medicine, National Taiwan University, Taipei, Taiwan; e Department of Pharmacy, National Taiwan University Cancer Center, Taipei, Taiwan; f Graduate Institute of Clinical Pharmacy, National Taiwan University College of Medicine, Taipei, Taiwan; Tainan Hospital, Department of Health, Executive Yuan

**Keywords:** vancomycin resistant enterococcus, daptomycin, infective endocarditis, pharmacokinetics, animal model

## Abstract

A high daptomycin dose has been suggested for treating vancomycin-resistant Enterococcus faecium (VREf) infections. However, even a 12 mg/kg daptomycin dose might be insufficient for treating VREf with high daptomycin minimum inhibitory concentrations (MICs). Additionally, animal pharmacodynamic and infection models to confirm the efficacy of 12 mg/kg daptomycin are lacking. Male Wistar rats were used for pharmacokinetic profiling and for the development of an infective endocarditis (IE) model. Daptomycin-susceptible dose-dependent VREf (DSE) (MIC of 0.5 mg/L) and daptomycin nonsusceptible VREf (DNSE) (MIC of 8 mg/L) were used for the IE models. The bacterial load of vegetation was the primary outcome and was evaluated after 3 days of daptomycin treatment. Daptomycin administered subcutaneously (s.c.) at 45 and 90 mg/kg, which corresponded to maximum serum concentrations (*Cmax*) of 122.6 mg/L and 178.5 mg/L, respectively, was equivalent to doses of 8 mg/kg and 12 mg/kg, respectively, in humans. The *Cmax*/MIC value was correlated with the bacterial load of vegetation after treatment (*r* = −0.88, *P* < 0.001). The 90 mg/kg s.c. group showed a significantly lower bacterial load of vegetation (log_10_ CFU/g) than the 45 mg/kg s.c. group against DSE (0 versus 4.75, *P* < 0.001) and DNSE (5.12 versus 6.98, *P* = 0.002). The 90 mg/kg s.c. group did not sterilize the vegetation against DNSE. Although the human equivalent dose of 12 mg/kg daptomycin was more effective than the smaller dose in reducing the bacterial load in DSE and DNSE IE, the dose could not sterilize the vegetation during a DNSE treatment. Further treatment strategies by which to manage severe VREf infections, especially at high daptomycin MICs, are urgently needed.

**IMPORTANCE** Using a rat IE model with pharmacokinetic analysis, the treatment response of VREf IE was found to be daptomycin dose-dependent, presented as *Cmax*/MIC or as the 24 h area under the concentration-time curve (AUC_0–24_)/MIC. Daptomycin 90 mg/kg s.c. significantly reduced the bacterial load against DSE and DNSE. It also showed significant activity against DSE and DNSE, compared to 45 mg/kg s.c. Although daptomycin 90 mg/kg can eradicate the bacterial load after 3 days of treatment against DSE, eradication cannot be achieved with 90 mg/kg daptomycin against DNSE.

## INTRODUCTION

Vancomycin-resistant (VR) enterococci (VRE) were first reported in 1986 (1), and they have since been increasingly implicated in hospital-acquired infections, especially those in patients admitted to intensive care units (2). VRE bloodstream infection (BSI) is a significant independent predictor of mortality in patients with enterococcal BSI (3), and the current treatment options are limited (4, 5). Daptomycin is an attractive treatment agent, as it exhibits concentration-dependent bactericidal activity (6) and rapid bactericidal activity against enterococci (7). It is increasingly being used in the treatment of VRE-BSI (8). The 24 h area under the concentration-time curve (AUC_0–24_)/minimum inhibitory concentration (MIC) ratio or the maximum serum concentration (*Cmax*)/MIC ratio is the pharmacodynamic parameter that best correlates with the *in vivo* efficacy of daptomycin (9). High daptomycin doses are associated with increases in AUC_0–24_ and *Cmax* (10). Recent studies have shown that daptomycin administered at high doses is associated with better outcomes of VRE-BSI (11–13), compared to that administered at customary doses. Experts suggest using a daptomycin dose of up to 12 mg/kg/day for complicated VRE-BSI and endocarditis (14). The Clinical and Laboratory Standards Institute (CLSI) also suggested treating E. faecium bacteremia with 8 to 12 mg/kg daptomycin (15). VRE isolates with a high daptomycin MIC, although susceptible, were associated with increased microbiological failure (16). Considering the MIC distribution of E. faecium (17), there remain concerns that even a dose of 12 mg/kg may be insufficient to treat an infection caused by E. faecium with a high MIC (18, 19).

Enterococci are inherently less virulent organisms that generally infect immunocompromised patients. The risk factors for VRE infections may affect survival, which complicates the evaluation of treatment responses to antibiotics (2). Animal models have critically contributed to our understanding of the pathogenesis of enterococcal infections (20). A rodent model can be used to evaluate antimicrobial efficiency (20). Infective endocarditis (IE) is a special form of VRE-BSI that is characterized by a high bacterial burden. A high VRE inoculum compromises the efficacy of daptomycin (21). A rat IE model can be used to study bacterial virulence and the response to antimicrobial agents, and the data of such studies are reproducible (20). A few animal studies have compared the efficacy of different doses of daptomycin against *Enterococcus* (22, 23). However, few animal studies have evaluated the efficacy of 12 mg/kg daptomycin against VR E. faecium (24).

Since daptomycin exhibits concentration-dependent bacterial killing, we hypothesized that a dose simulating the human dose of 12 mg/kg daptomycin will lead to better bacterial clearance in a rat IE model than will a lower dose. Since different doses and routes of administration of daptomycin have been used in the *Enterococcus* IE model (22, 23, 25–31), we further examined the pharmacokinetics of the different routes and doses used in this study to better simulate doses that are administered to humans. Drugs might be eliminated faster, especially in a small rodent pharmacokinetic model (32). However, daptomycin was given as a human dose intravenously (i.v.) in some animal studies (33). We also demonstrate that the pharmacokinetics of 12 mg/kg daptomycin i.v. in rats is different from the pharmacokinetics of 12 mg/kg daptomycin i.v. in humans.

## RESULTS

### Selected isolates.

Two clinical isolates were selected for the rat IE model analysis. Both isolates were resistant to vancomycin, with MICs > 128 mg/L. Isolate NTUH664 was a daptomycin-susceptible, dose-dependent E. faecium (DSE) and was observed to have a daptomycin MIC of 0.5 mg/L. Isolate NTUH688 was a daptomycin nonsusceptible E. faecium (DNSE), with a daptomycin MIC of 8 mg/L.

### Pharmacokinetics.

**FIG 1:**
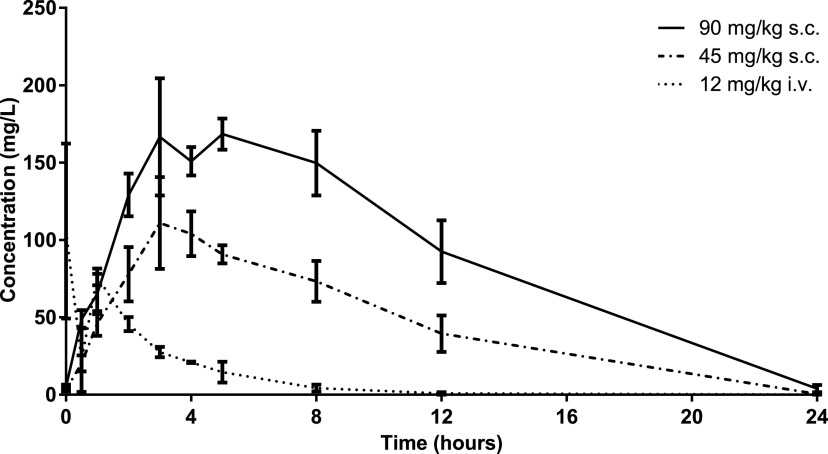
Concentration-time profile of different daptomycin regimens in a rat model. Plots show values from individual rats and the mean ± standard deviation (SD).

The pharmacokinetic profiles of the different daptomycin regimens in our rat model are presented in Table 1 and Fig. 1. We showed that after a single dose of daptomycin at 12 mg/kg i.v., 45 mg/kg subcutaneously (s.c.), and 90 mg/kg s.c., the mean *Cmax* values were 106.2, 122.6, and 178.5 mg/L. The mean AUC_0–24_ (mg × h/L) was significantly lower in the 12 mg/kg i.v. group than in the other two groups.

**TABLE 1:** Pharmacokinetic parameters of daptomycin after a single subcutaneous administration in rats

Dose (mg/kg), route	*Cmax*^*c*^ (mg/L)	*T*_max_^*d*^ (h)	AUC_0–24_^*e*^ (mg × h/L)	*t*_1/2_^*f*^ (h)
Rat in this study				
12, i.v.^*a*^	106.2 (56.2)	0.3 (0.6)	228.2 (15.7)	4.6 (0.9)
45, s.c.^*b*^	122.6 (17.1)	3.3 (0.6)	986.7 (185.6)	3.0 (1.3)
90, s.c.^*b*^	178.5 (19.7)	3.3 (0.6)	2,053.6 (352.3)	2.9 (0.4)
Human (34)				
8, i.v.^*a*^	123.3 (13.0)		858.2 (24.9)	
12, i.v.^*a*^	183.7 (13.6)		1,277.4 (19.8)	

a i.v., intravenously.

b s.c., subcutaneously.

c *Cmax*, maximum serum concentration.

d *T*_max_, time required to reach *Cmax*.

e AUC_0–24_, area under the concentration-time curve from 0 to 24 h.

f *t*_1/2_, half-life.

### IE models.

**FIG 2:**
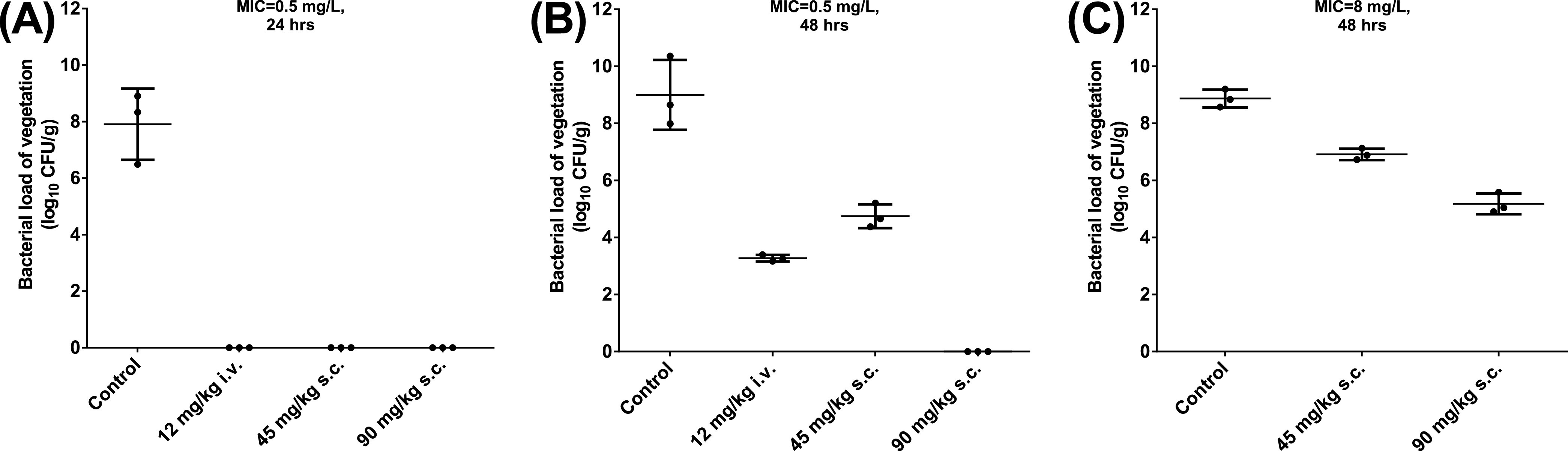
Bacterial load of vegetation after different daptomycin regimens in a rat infective endocarditis model. Panel (A) represents Enterococcus faecium with a daptomycin minimum inhibitory concentration (MIC) of 0.5 mg/L and 24 h from inoculation to treatment. Panel (B) shows a daptomycin MIC of 0.5 mg/L and 48 h from inoculation to treatment. Panel (C) shows a daptomycin MIC of 8 mg/L and 48 h from inoculation to treatment. The scatterplots show values from individual rats and the mean ± standard deviation (bars).

60 rats underwent the IE model experiments. The IE model analysis included 33 rats that survived the entire therapy course and had a transaortic valve catheter in the appropriate position. 4 rats were excluded because their transaortic valve catheters were not in the appropriate position. 23 rats died during the course of therapy (Table 2 and 3). There were no significant survival differences compared between the different VRE isolates, daptomycin doses, and treatment routes. The responses to the different daptomycin regimens and the different durations from inoculation to treatment are shown in Table 2 and Fig. 2.

**TABLE 2:** Comparison of different daptomycin regimens for a daptomycin-susceptible, dose-dependent Enterococcus faecium infective endocarditis rat model

Time from inoculation to treatment (h)	Dose (mg/kg), route	Bacterial load of vegetation (log_10_ CFU^*c*^/g)	Rate of sterile vegetation	Bacterial load in blood (log_10_ CFU^*c*^/mL)	*Cmax*^*d*^ (mg/L)	AUC_0–24_^*e*^ (mg × h/L)	No. of rats surviving^*j*^/no. treated
24	12, i.v.^*a*^	0 (0)	100	0 (0)	106.2 (56.2)	228.2 (15.7)	3/6
24	45, s.c.^*b*^	0 (0)	100	0 (0)	122.6 (17.1)	986.7 (185.6)	3/4
24	90, s.c.^*b*^	0 (0)	100	0 (0)	178.5 (19.7)	2,053.6 (352.3)	3/4
24	Control	7.91 (1.26)^*f*^	0	3.67 (0.90)			3/4
48	12, i.v.^*a*^	3.27 (0.11)	0	0 (0)	106.2 (56.2)	228.2 (15.7)	3/7
48	45, s.c.^*b*^	4.75 (0.42)^*g*^	0	0 (0)	122.6 (17.1)	986.7 (185.6)	3/6
48	90, s.c.^*b*^	0 (0)^*h*^^,^^*i*^	100	0 (0)	178.5 (19.7)	2,053.6 (352.3)	3/5
48	Control	9.00 (1.22)	0	5.02 (5.19)			3/6

a i.v., intravenously.

b s.c., subcutaneously.

c CFU, colony forming units.

d *Cmax*, maximum serum concentration.

e AUC_0–24_, area under the concentration-time curve from 0 to 24 h.

f *P* = 0.34, compared between 24 and 48 h from inoculation to treatment.

g *P* = 0.005, compared between 45 mg/kg and the control group.

h *P* < 0.001, compared between 90 mg/kg and the control group.

i *P *< 0.001, compared between 90 and 45 mg/kg subcutaneously.

j Survived the entire therapy.

**TABLE 3:** Comparison of different daptomycin regimens for daptomycin nonsusceptible Enterococcus faecium infective endocarditis rat model

MIC^*a*^ (mg/L)	Dose (mg/kg), route	Bacterial load of vegetation (log_10_ CFU^*c*^/g)	Rate of sterile vegetation	Bacterial load in blood (log_10_ CFU^*c*^/mL)	*Cmax*^*d*^ (mg/L)	AUC_0–24_^*e*^ (mg × h/L)	No. of rats surviving^*i*^/no. treated
8	45, s.c.^*b*^	6.92 (0.20)^*f*^	0	0.75 (1.30)	122.6 (17.1)	986.7 (185.6)	3/3
8	90, s.c.^*b*^	5.18 (0.36)^*g*^^,^^*h*^	0	0 (0)	178.5 (19.7)	2,053.6 (352.3)	3/4
8	Control	8.87 (0.31)	0	2.22 (1.92)			3/7

a MIC, minimum inhibitory concentration.

b s.c., subcutaneously.

c CFU, colony forming units.

d *Cmax*, maximum serum concentration.

e AUC_0–24_, area under the concentration-time curve from 0 to 24 h.

f *P *< 0.001, compared between 45 mg/kg and the control group.

g *P *< 0.001, compared between 90 mg/kg and the control group.

h *P* = 0.002, compared between 90 and 45 mg/kg subcutaneously.

i Survived the entire therapy.

Among the groups that received the first dose of treatment after 24 h of DSE inoculation, the control group showed a mean bacterial load of 7.91 log_10_ colony forming units (CFU)/g, whereas all three treatment groups, despite their different *Cmax* and AUC_0–24_ values, showed undetectable bacterial loads of vegetation (compared to the control group, all *P* < 0.001).

Among the groups that received the first dose of treatment after 48 h of DSE inoculation, the control group showed a bacterial load of vegetation similar to that of the 24 h group (*P = *0.34). The 45 mg/kg s.c. group showed significant differences in bacterial load of vegetation compared to the control group (*P = *0.005). The 90 mg/kg s.c. group had a significantly lower bacterial load of vegetation than did the control group (*P* < 0.001) and the 45 mg/kg s.c. group (*P < *0.001).

The pharmacokinetic parameters of 12 mg/kg daptomycin i.v. in rats were significantly different from those in humans. When the first dose of daptomycin was administered after 24 h of inoculation, the treatment responses in the different treatment groups could not be distinguished. Therefore, we compared the treatment responses in groups with different daptomycin MICs by administering different daptomycin doses in the IE model, in which the first dose of daptomycin was administered 48 h after inoculation.

There were no significant differences observed between the DSE and DNSE control groups (*P* = 0.87). Among the DNSE group, the 45 mg/kg s.c. and 90 mg/kg s.c. treatments significantly reduced the bacterial load compared to that observed in the control group (both *P* < 0.001). The 90 mg/kg s.c. treatment group also showed a significantly lower bacterial load than did the 45 mg/kg s.c. group (*P* = 0.002) (Table 3).

### The correlation between pharmacodynamic parameters and treatment response.

**FIG 3:**
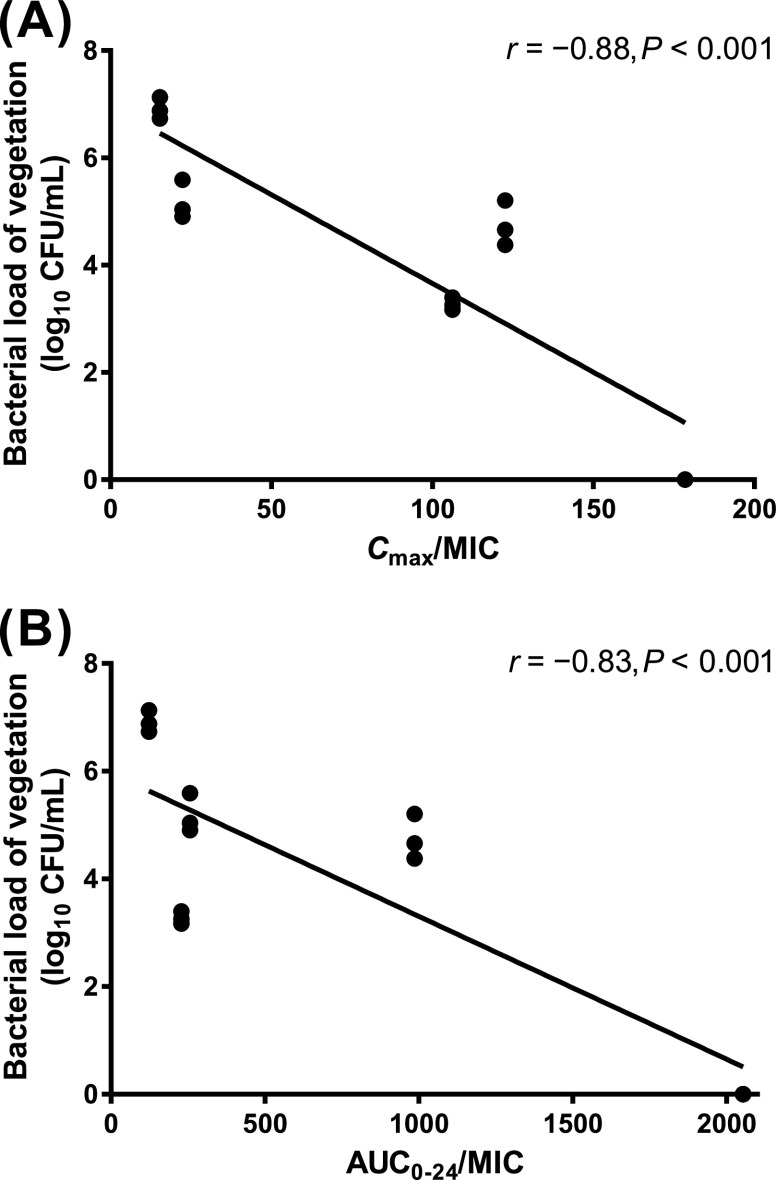
Correlation between the bacterial load of vegetation and (A) *Cmax*/MIC and (B) AUC_0–24_/MIC. The solid line represents the linear regression fitted line. AUC_0–24_, area under the concentration-time curve from 0 to 24 h. CFU, colony forming units. *Cmax*, maximum serum concentration. MIC, minimum inhibitory concentration.

The correlation between the pharmacodynamic (PD) parameters (i.e., *Cmax*/MIC and AUC_0–24_/MIC) and the bacterial load, determined using the IE model, in which the first dose of daptomycin was administered after 48 h of inoculation, is shown in Fig. 3. Both the *Cmax*/MIC value and the AUC_0–24_ value were significantly correlated with the bacterial load of vegetation after treatment (*r* = −0.88, *P* < 0.001 and *r* = −0.83, *P* < 0.001, respectively).

## DISCUSSION

In the current study, the treatment response was found to be daptomycin dose-dependent, presented as *Cmax*/MIC or AUC_0–24_/MIC. Daptomycin 90 mg/kg s.c. significantly reduced the bacterial load against DSE and DNSE. It also displayed significant activity against DSE and DNSE, compared to that displayed by 45 mg/kg s.c. Although daptomycin 90 mg/kg can eradicate the bacterial load after 3 days of treatment against DSE, eradication cannot be achieved with 90 mg/kg daptomycin against DNSE.

The mean *Cmax* was 123.3 mg/L for a daily dose of 8 mg/kg and 183.7 mg/L for 12 mg/kg in healthy volunteers (34). Therefore, 45 mg/kg s.c. and 90 mg/kg s.c might represent human doses of 8 mg/kg and 12 mg/kg, respectively (34). The pharmacodynamic parameters that best describe the efficacy of daptomycin are *Cmax*/MIC and AUC_0–24_/MIC (9). Since the efficacy was much more pronounced in the infrequently administered dosing regimens, *Cmax*/MIC is likely the most predictive (35, 36). The selected rat dose from which to simulate the human dose is slightly different from the others (37–39). The differences might be due to the differences in routes (38, 39), rat strains (37), and drug concentration measurement methods (37–39). Computer-controlled continuous infusion might best mimic human pharmacokinetics; however, the system works best for antimicrobials with time-dependent activities (35).

We evaluated the treatment responses of different daptomycin regimens after 48 h of delayed treatment in the E. faecium IE model (23). In most Enterococcus IE models, the first dose of daptomycin was administered 24 h after inoculation (22, 25, 28, 29). However, by using a 24 h delay, we showed that the treatment responses of different daptomycin regimens could not be differentiated. A possible explanation is that E. faecalis has been used in previous studies (22, 25, 28, 29). E. faecalis produces biofilms more often than does E. faecium, and biofilm formation may be an important factor in the pathogenesis of enterococcal infections (35, 40). This argument is also supported by the fact that the inoculum of E. faecium was demonstrated to be higher than that of E. faecalis in previous infective endocarditis models (23, 30). However, this finding highlights the importance of early treatment. Even with inadequate PD parameters, the 12 mg/kg i.v. group eradicated the bacteria in the vegetation.

Daptomycin exhibits concentration-dependent bactericidal activity (6). Increased daptomycin doses are associated with increased AUC_0–24_ and *Cmax* values (10). An increased daptomycin dose has been shown to be related to better outcomes in patients with VRE-BSI (11–13). Expert opinion suggests that a daptomycin dose of up to 12 mg/kg might be used for the treatment of complicated VRE-BSI or VRE endocarditis (14). CLSI also suggested using ≤4 mg/L as a breakpoint of dose-dependent susceptibility and using a dose of 8–12 mg/kg for serious infections caused by E. faecium (15). However, even with doses of 12 mg/kg, it is not possible to treat infections caused by isolates at the upper end of the wild-type distributions of E. faecium (with MICs of 4 or 8 mg/L) (19). The present study supports this concern. We showed that the treatment response was highly correlated with *Cmax*/MIC and with AUC_0–24_/MIC. We also showed that a human equivalent dose of 12 mg/kg (90 mg/kg s.c. in rats) exhibited a significant bacterial load reduction compared to a human equivalent dose of 8 mg/kg (45 mg/kg s.c. in rats) in treating DNSE (*P* < 0.001). However, sterile vegetation even under 90 mg/kg s.c. was only achieved for the DSE, not for the DNSE IE. When treating DNSE IE, a substantial bacterial load of vegetation remained, even after 3-days of 90 mg/kg s.c. treatment. *Cmax* and AUC were highly correlated with daily doses in humans and rodents (10, 36). In order to achieve the same PD target (i.e., *Cmax*/MIC or AUC/MIC) for VR E. faecium with MIC = 0.5 and 8 mg/L, the daily dose is supposed to be 16-fold higher, which might be inapplicable. The increase in the daily dose proportionally increases the trough concentration (10), which is correlated with the side effects of rhabdomyolysis (41). If we considered the AUC_0–24_/MIC as the reference of simulated human doses, then a dose of 90 mg/kg s.c. is even higher than a human dose of 12 mg/kg. The efficacy of high dose daptomycin in treating VRE BSI might be more questionable.

This study had several limitations. First, the sample size was limited. The sample size calculation was based on the bacterial load of a previous study (23) and on the assumption that the clinically significant bacterial load reduction was a 100-fold decrease. We confirmed the hypothesis that 90 mg/kg s.c. was more effective than 45 mg/kg s.c. in reducing the bacterial load of DNSE IE. A sample size that is too large will lead to the unnecessary waste of resources and animals (42). However, results with no significant differences should be interpreted with caution. That is to say, from the point of view of sterile vegetation, the facts of whether 45 mg/kg s.c. is sufficient for treating DSE IE and whether 90 mg/kg s.c. is better than 45 mg/kg s.c. for treating DSE IE was not addressed in the present study. Second, the PK parameters were evaluated in uninfected rats, and whether the infected rat IE model would present the same PK parameters remains unknown. Third, understanding the roles of other treatment approaches, such as combination treatment (43, 44), and whether they can decrease the PD target and improve the outcome warrants further study. Lastly, the blood culture was not taken after inoculation to ensure bacteremia. However, the nondifferential misclassification might bias the results toward the null (45).

In conclusion, using the established rat IE model, we showed that 90 mg/kg s.c., which simulated a human dose of 12 mg/kg, is more effective than 45 mg/kg s.c. in reducing the bacterial load of DSE and even that of DNSE IE. There was a dose-dependent treatment response between the bacterial load and the different daptomycin regimens. However, sterile vegetation could not be achieved at a human equivalent dose of 12 mg/kg when treating DNSE, and a substantial bacterial load of vegetation remained. Further treatment strategies by which to manage severe VR E. faecium infections, especially those with high daptomycin MICs, are urgently needed.

## MATERIALS AND METHODS

### Bacterial isolates and antimicrobial susceptibility testing.

Blood culture isolates were obtained from a clinical microbiology laboratory. *Enterococcus* spp. were identified using the Vitek 2 identification system (bioMérieux Inc., La Balme les Grottes, France). The MIC of vancomycin was determined using a Sensititre GPN3F (Trek Diagnostics, West Sussex, England). Vancomycin-resistant *Enterococcus* was defined as an *Enterococcus* isolate with a vancomycin MIC ≥ 32 mg/L. Two clinical VR E. faecium isolates (with daptomycin MICs of 8 mg/L and 0.5 mg/L) were used in the study. The daptomycin MIC was measured using the broth microdilution method. We used cation-adjusted Mueller-Hinton broth (Becton, Dickinson, Le Pont-de-Claix, France) supplemented with 50 μg/mL calcium. The MIC breakpoints were based on the CLSI criteria of interpretation (15).

### IE model.

Approval for animal use was obtained from the National Taiwan University Institutional Animal Care and Use Committee before the commencement of the experiments. Endocarditis was induced in male Wistar rats weighing ~330 g by using previously developed methodologies with slight modifications (46). To expose the right carotid artery for the placement of an intravascular catheter, the animals were anesthetized with 1 μL/g of Zoletil 50 (1 mg/mL) plus Rompun (23.32 mg/mL). A sterile polyethylene catheter (Intramedic PE-10; Clay Adams, Parsippany, NJ, USA) was inserted through a small incision and advanced 4 cm into the left ventricle. The catheter was ligated and left in place for the duration of the experiment. 24 h after the catheter was placed across the aortic valve, the bacterial inoculum, which was presuspended in saline, and 1 mL of 10^8^ CFU/mL of VRE were administered intravenously via the tail vein. We evaluated the actual inoculum based on the CFU count. The rats received antibiotic treatment at 24 h or 48 h after the bacterial inoculation.

### Antimicrobial therapy for the endocarditis model.

Daptomycin at doses of 45 mg/kg/day or 90 mg/kg/day, s.c. was used to simulate a conventional or high daptomycin dose in humans, respectively (34, 37, 38). In addition, 12 mg/kg/day daptomycin i.v. was used for a comparison. Preliminary data showed that the pharmacokinetic (PK) parameters of 12 mg/kg daptomycin i.v. in rats were significantly different from those in humans. Therefore, 12 mg/kg i.v. in rat was only given for DSE but not for DNSE. The animals were treated for 3 days. A group of infected but phosphate-buffered saline-treated rats was used as a control. 24 h after the last treatment, the animals were sacrificed via anesthetic overdose. The aortic valve vegetation and 1 mL of blood were obtained aseptically from the sacrificed animals, weighed, and homogenized in 1 mL of phosphate-buffered saline. Serial dilutions of homogenized tissues were performed, and 100 μL of each dilution, including the undiluted sample, were plated on brain heart infusion agar to enumerate the bacteria. The animals were included in the final analysis only if they survived the entire therapy and if their catheters were found across the aortic valve in the left ventricle. The detection limit of the bacterial load of vegetation was 2 log_10_ CFU/g.

### Pharmacokinetics.

To ensure that the pharmacokinetic parameters in the animal models were similar to those in humans (34), daptomycin (45 and 90 mg) was administered as a single s.c. dose, and 12 mg daptomycin was also administered as a single i.v. dose, to healthy male Wistar rats, weighing ~330 g. The experiments were performed in triplicate. Blood samples were collected from the inferior vena cava at 0, 0.5, 1, 2, 3, 4, 5, 8, 12, and 24 h in heparinized tubes. Plasma was collected via centrifugation, and the samples were stored at −80°C until analysis.

A daptomycin quantification assay was performed as previously described, using an ultra high performance liquid chromatography (UHPLC)-electrospray ionization (ESI)-tandem mass spectrometry (MS/MS) analysis (47). Daptomycin was purchased from Glentham Life Sciences (Corsham, UK), and ^2^H_5_-daptomycin trifluoroacetic acid was purchased from Alsachim (Illkirch Graffenstaden, France). All of the UHPLC-ESI-MS/MS analyses were performed using an Agilent 1290 UHPLC system coupled with an Agilent 6460 triple quadrupole mass spectrometer (Agilent Technologies, Santa Clara, CA, USA).

### Statistical analysis.

We hypothesized that a daptomycin dose greater than the conventional dose can effectively lower the bacterial load in vegetation by 100-fold. According to findings from a previous study on an *Enterococcus* rat IE model (23), group sample sizes of 3 and 3 can achieve 80% power to detect a difference of 2 log_10_ CFU/mL between the null hypothesis that both group means are 6.5 log_10_ CFU/mL and the alternative hypothesis that the mean of the group with a higher dose is 4.5 log_10_ CFU/mL, with estimated group standard deviations of 0.8 and 0.8 and a significance level (α) of 0.05, using a one-sided two-sample *t* test (42).

Correlations between the pharmacodynamic parameters of daptomycin and treatment responses were examined using Pearson’s correlation coefficient. Percentages were calculated for the categorical variables. For the continuous variables, the mean and the standard deviation were calculated, and two-sample *t* tests were used for the comparisons. All analyses were performed at a two-tailed significance level of 0.05. All of the statistical analyses were conducted using the Stata software package (v. 17; StataCorp, College Station, TX, USA).

### Ethics approval.

This study was approved by the Research Ethics Committee and the Institutional Animal Care and Use Committee of our institute in accordance with the relevant and appropriate guidelines (20180312).

### Data availability.

The data are available as Supplemental Material.
